# Comparison of the Effects of Different Manual Therapy Techniques on Mandibular Mobility, Cervical Joint Position Sense, Jaw Function and Anxiety Levels in Individuals With Rheumatoid Arthritis and Temporomandibular Disorders: A Randomized Controlled Trial

**DOI:** 10.1111/joor.70218

**Published:** 2026-05-23

**Authors:** Kübra Aktaş, Hakan Polat, Tuba Maden, Burhan Fatih Koçyiğit

**Affiliations:** ^1^ Graduate Education Institute, Master's Program in Physiotherapy and Rehabilitation SANKO University Gaziantep Turkey; ^2^ Department of Physiotherapy and Rehabilitation, Faculty of Health Sciences SANKO University Gaziantep Turkey; ^3^ Department of Physiotherapy and Rehabilitation, Faculty of Health Sciences Gaziantep University Gaziantep Turkey; ^4^ Department of Physical Medicine and Rehabilitation, Adana City Training and Research Hospital University of Health Sciences Adana Turkey

**Keywords:** anxiety, joint mobilization, manual therapy, proprioception, rheumatoid arthritis, soft tissue mobilization, temporomandibular disorders

## Abstract

**Objective:**

This study aimed to compare the effects of soft tissue mobilization (STM) and joint mobilization (JM) on mandibular mobility, cervical joint position sense (JPS), jaw function and anxiety levels in individuals with rheumatoid arthritis (RA) and temporomandibular disorders (TMD).

**Methods:**

This randomized controlled study included 57 patients with RA and TMD, who were allocated to three groups: STM group (*n* = 19), JM group (*n* = 19) and control group (CG; *n* = 19). Interventions were applied twice weekly for 6 weeks. Outcome measures included mandibular range of motion (ROM), jaw function assessed using the Jaw Functional Limitation Scale‐20 (JFLS‐20), cervical JPS using a CROM device, and anxiety levels using the Generalized Anxiety Disorder‐7 (GAD‐7) questionnaire. Statistical significance was set at *p* < 0.05.

**Results:**

Both intervention groups demonstrated significant improvements in mandibular mobility, jaw function, and anxiety compared to CG (*p* < 0.05), whereas the CG showed deterioration in mouth opening, protrusion and anxiety over time. In mandibular mobility, both STM and JM showed significant improvements across most parameters, with JM showing a more consistent pattern of improvement. Significant improvements in cervical JPS were observed in the STM group for flexion, extension, and left rotation, and in the JM group for flexion only. No significant differences were found between the intervention groups in jaw function or anxiety outcomes.

**Conclusion:**

Soft tissue mobilization enhances cervical proprioception more effectively, whereas joint mobilization provides greater improvements in mandibular mobility. Both manual therapy techniques are effective, non‐invasive and clinically applicable options for managing TMD in individuals with RA.

**Trial Registration:**

ClinicalTrials.gov: NCT07171671

## Graphical abstract

Joint mobilization and soft tissue mobilization improved mandibular mobility, cervical proprioception, jaw function and anxiety levels in rheumatoid arthritis patients with temporomandibular disorders compared to the control group.

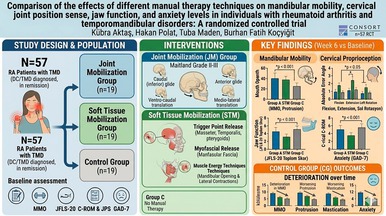

## 1 Introduction

Rheumatoid arthritis (RA) is a chronic, progressive systemic inflammatory disease characterized by synovial joint involvement and a polyarticular course. RA affects not only bone structures but also periarticular soft tissues, the synovial membrane and surrounding muscles, resulting in complex functional limitations [1, 2, 3]. While RA primarily involves the joints of the hands and feet symmetrically, it can also affect the temporomandibular joint (TMJ), albeit less frequently [2]. Individuals with RA are 2.5 times more likely to develop temporomandibular disorders (TMD) compared with healthy populations [4]. TMJ involvement may contribute to musculoskeletal dysfunction by affecting the masticatory muscles, joint surfaces and surrounding tissues [5, 6]. TMD is typically associated with symptoms of orofacial and neck pain, edema, crepitus, limitation of maximal mouth opening (MMO) and stiffness in jaw movements [2, 7]. Individuals with TMD may demonstrate alterations in cervical joint position sense (JPS) [8]. Structural changes such as condylar erosion, disc thickening or displacement, synovial membrane hypertrophy, and occlusal alterations have been frequently observed in patients with RA using advanced imaging modalities. Recent studies employing cone‐beam computed tomography indicate that these structural alterations correlate with disease duration and severity [9, 10]. Psychological factors, including anxiety, have also been associated with TMD and may influence symptom severity and functional limitations, supporting their assessment in clinical studies [11].

Conservative interventions, including therapeutic exercises, occlusal splints, laser therapy, transcutaneous electrical nerve stimulation (TENS), manual therapy and intra‐articular injections, are commonly used to manage TMD in rheumatic diseases. These approaches are non‐invasive, reversible, and primarily aim to alleviate pain and improve function [12, 13, 14]. Recent systematic reviews and meta‐analyses have shown that multimodal rehabilitation protocols combining manual therapy, therapeutic exercise, and electrotherapy can effectively reduce pain and improve mandibular range of motion (ROM) [15]. Manual therapy represents a conservative approach to functional recovery through several physiological mechanisms, including enhanced local circulation, improved proprioceptive feedback, reduction of muscle hyperactivity, and increased joint mobility. However, evidence regarding the use of manual therapy in the RA population remains limited. Most existing studies have focused on individuals with TMD in the general population, whereas interventions for patients with RA have primarily emphasized pharmacological management, joint injections, or laser therapy [12].

Recent systematic reviews and meta‐analyses have reported TMJ mobilization is associated with significant reductions in pain intensity and improvements in maximum mouth opening (MMO) in patients with TMD, with effects generally observed immediately after treatment and peaking within several weeks of intervention [16]. Earlier systematic reviews further support the beneficial effects of manual therapy in TMD, reporting improvements in pain, mandibular mobility, and self‐reported disability compared with control interventions. However, these reviews commonly evaluate multimodal approaches that combine mobilization, soft tissue techniques, exercise, and massage, making it difficult to isolate the specific contribution of TMJ mobilization alone [15, 17, 18]. In patients with rheumatic diseases, including RA, rehabilitation‐based approaches such as manual therapy and therapeutic exercise have also been shown to improve pain and functional outcomes in TMJ involvement, although the evidence remains heterogeneous and primarily derived from conservative treatment studies rather than isolated interventions [12].

Despite growing interest in conservative treatment strategies for TMD, current evidence remains insufficient to determine the relative effectiveness of available interventions because of the multifactorial nature of TMD and the heterogeneity of treatment approaches. Findings from recent meta‐analyses emphasize the need for further well‐designed comparative clinical trials to evaluate different conservative therapies and support the development of targeted, evidence‐based treatment protocols for distinct TMD presentations [16].

Joint mobilization is a manual therapy approach used to address capsular restrictions and intra‐articular changes in the TMJ. Techniques aim to restore functional range, normalize joint biomechanics, reduce excessive compression, and promote lubrication through synovial fluid, typically involving traction or translational movements in anteroposterior, mediolateral or caudal‐ventral directions. Soft tissue mobilization, also a commonly used manual therapy approach, targets secondary muscle hyperactivity and myofascial tension associated with TMD. Interventions focus on muscles frequently affected in TMD, including the lateral pterygoid, masseter, and temporalis, using massage, stretching and relaxation techniques to relieve pain, reduce spasm, and improve tissue mobility [16]. Given that RA‐related TMD involves both intra‐articular structural alterations and secondary myofascial adaptations, these two approaches represent distinct but complementary therapeutic targets. However, previous studies have predominantly evaluated multimodal interventions, limiting the ability to determine the individual contribution of specific techniques. Direct comparative evidence between joint‐focused and soft tissue‐focused manual therapy approaches remains limited, particularly in patients with RA‐related TMD. Therefore, comparing these two commonly used yet mechanistically different manual therapy techniques may help clarify their relative contributions to functional outcomes and support more targeted, evidence‐based clinical decision‐making.

In this context, the present study aimed to comparatively evaluate the effectiveness of two manual therapy approaches, joint mobilization and soft tissue mobilization, on mandibular mobility, jaw function, and cervical JPS, in individuals with RA and TMD, while also assessing anxiety as a secondary outcome. This randomized controlled study addresses a significant gap by evaluating manual therapy interventions specifically for TMD in patients with RA and aims to provide clinically relevant evidence to inform conservative management strategies.

## 2 Methods

### 2.1 Study Design

This was a three‐arm, parallel‐group, randomized controlled clinical study. The study adhered to the principles of the Declaration of Helsinki and received approval from the Scientific Research Ethics Committee of Adana City Training and Research Hospital (approval no: 147/5, date: 11.09.2024). The study was registered on ClinicalTrials.gov (NCT07171671). Written informed consent was obtained from all participants prior to enrolment. The study was reported according to the Consolidated Standards of Reporting Trials (CONSORT) statement [19].

### 2.2 Study Setting and Participants

The study was conducted at the Physical Therapy and Rehabilitation Unit of Adana City Training and Research Hospital between October 2024 and May 2025. Participants were recruited from patients presenting to the Rheumatology and Physical Therapy clinics of the same institution. Eligible participants were adults diagnosed with RA according to the ACR/EULAR 2010 criteria [20] and TMD based on the DC/TMD criteria [21]. Participants were referred by a rheumatologist, who confirmed remission status at the time of inclusion based on clinical evaluation. The study sample consisted of patients presenting with clearly defined TMD symptoms, including pain in the TMJ region, discomfort during mastication, limited mouth opening, joint sounds, and tenderness of the masticatory muscles. Inclusion criteria were age between 18 and 65 years, chronic‐phase RA, a confirmed diagnosis of TMD, and adequate cognitive function. Exclusion criteria were a history of maxillofacial trauma or surgery, orthodontic treatment within the last 6 months, presence of trigeminal neuralgia or facial nerve paralysis, any rheumatic disease other than RA, use of intraoral splints or dentures, missing molar teeth, and a history of manual therapy, injections, or other interventions targeting the cervical or TMJ regions within the last 6 months. Discontinuation criteria comprised irregular attendance to therapy sessions, voluntary withdrawal from the study, or initiation of orthodontic treatment during the study period. A total of 60 individuals were enrolled and randomly allocated to three groups (*n* = 20 per group): Soft Tissue Mobilization (STM) group, Joint Mobilization (JM) group and Control Group (CG).

### 2.3 Sample Size

The sample size was calculated based on a previous randomized controlled trial on manual therapy in patients with TMD [22], using the mean MMO score, with a significance level of *α* = 0.05 and a power of 0.80. This calculation indicated that a minimum of 54 participants were required, with at least 18 individuals per group.

### 2.4 Randomization and Blinding

Randomization and allocation concealment were implemented through a centralized, web‐based system (www.randomizer.org) using a computer‐generated random sequence. To minimize selection bias, the sequence was managed by an independent researcher who was not involved in participant recruitment, clinical assessment, or treatment procedures. Group allocation was concealed from all investigators involved in enrolment and assessment and was disclosed only after participants had been registered and baseline data were entered into the system. This ensured that treatment assignment remained inaccessible to the study team until the moment of allocation.

### 2.5 Study Procedures

Prior to intervention, demographic data were collected from all participants using a Demographic Information Form. To protect personal data, all information was anonymized and participants were assigned a unique study identification number.

Both before and after the intervention, participants were evaluated using the following assessments: cervical ROM and JPS were measured using a C‐ROM device; TMJ ROM was assessed using a millimetre ruler; muscle pressure pain thresholds were measured with an algometer; jaw function was assessed using the Jaw Functional Limitation Scale‐20 (JFLS‐20); and anxiety levels were evaluated using the Generalized Anxiety Disorder‐7 (GAD‐7) questionnaire.

Patients in the STM and JM groups received manual therapy sessions twice a week for 6 weeks, for a total of 12 sessions, with each session lasting 25–30 min. All manual therapy sessions were conducted one‐on‐one in the same environment (a quiet room at room temperature) by the same physical therapist.

In the STM group, therapy consisted of trigger point release, myofascial release, and muscle energy techniques (Appendix 1) [23].

Trigger point therapy was applied to the masseter, temporalis, digastric, medial pterygoid and lateral pterygoid muscles. Ischemic compression was applied for 60–90 s per trigger point, with pressure maintained at the patient's discomfort threshold but below the pain threshold until relaxation of the tissue was felt under the therapist's finger. This technique aimed to restore local circulation and relieve tension within the targeted muscles. Pressure was individualized for each patient and could be repeated on any tense muscle bands as needed. Myofascial release was performed on the masseter, temporalis, and digastric muscles along the muscle fibres from origin to insertion, with gradually increasing depth, and included release of the mandibular fascia [24]. To increase jaw mobility, muscle energy techniques were applied using isometric contraction followed by relaxation during mouth opening and lateral excursions. Trigger point therapy was applied to each muscle for 60–90 s in 3 sets, with 1–2 min of rest between sets. Myofascial release was applied for approximately 90 s, and muscle energy techniques were performed for 10 s per contraction in 3 sets, with 1–2 min of rest between sets.

In the JM group, TMJ mobilization was performed using caudal and anterior glides, ventro‐caudal translation, and medio‐lateral translation techniques (Appendix 2). Joint mobilization was applied with participants in a supine position, with relaxed jaw muscles and slightly parted lips. Techniques were performed according to Maitland classification at Grade II–III, using rhythmic oscillations without exceeding the patient's pain threshold. Caudal gliding aimed to widen the joint space by moving the condyle inferiorly, while anterior glide facilitated mandibular protrusion. Ventro‐caudal translation was applied to improve alignment of the joint surfaces, particularly during mouth opening. Medio‐lateral translation was performed to increase joint capsule elasticity and support lateral ROM. Caudal and anterior glides were applied for 30 s per set, 3 sets per session, with 1‐min rest between sets. Ventro‐caudal translation was applied for 10–20 s, 3 repetitions, and medio‐lateral translation for 10–15 repetitions. Each session lasted approximately 30 min [25].

The control group did not receive any manual therapy intervention and continued with their routine rheumatology follow‐up. A second evaluation was conducted 6 weeks after the baseline assessment. Upon completion of the study, participants in the control group were offered the option of a home exercise programme or referral for TMD‐specific treatment. All participants were monitored throughout the study for potential adverse events related to the interventions. No adverse events occurred in any group during the study period.

### 2.6 Assessments

Assessments were conducted twice, before and after treatment. To minimize bias, all assessments were performed by a physical therapist with 5 years of clinical experience who was not involved in the treatment of the participants. Due to the nature of the interventions, neither participants nor the treating therapist could be blinded.

TMD severity was evaluated at baseline using the Fonseca Anamnestic Index (FAI). FAI scores were used descriptively to characterize baseline TMD severity and were not included as an outcome measure in treatment‐effect analyses. The FAI is a self‐report questionnaire designed to evaluate both symptom severity and patient perception of TMD. The instrument consists of 10 items with three response options: yes (10 points), sometimes (5 points) or no (0 points). Based on the total score, TMD severity is classified as absent (0–15), mild (16–44), moderate (45–69) or severe (70–100) [26]. Reliability and validity of a Turkish version of the FAI have been demonstrated by Kaynak et al. [27].

Mandibular ROM was measured using a millimetre ruler. Participants were encouraged to open their mouth as widely as possible, even if mild discomfort was present. MMO was recorded as the linear distance in millimetres between the incisal edges of the upper and lower central incisors. Protrusion and retrusion were measured by recording the distance between the upper and lower central incisors during maximal forward and backward movement of the mandible. Lateral excursions to the right and left were assessed separately. The participant was asked to move the mandible laterally while the lines of contact between the upper and lower central incisors served as reference points. The lateral displacement was measured in millimetres to determine the range of lateral motion [28]. Three consecutive measurements were obtained and the mean values were used for analysis.

Cervical proprioception was assessed using a C‐ROM device (Baseline CROM 3; Fabrication Enterprises Inc., Elmsford, NY). Participants were seated upright, with the device secured on the head using the Velcro harness, and a magnetic reference aligned over the shoulders. Active cervical movements were performed in six directions: flexion, extension, right and left lateral flexion, and right and left rotation. For each movement, participants first reached the maximal range with eyes open. To assess proprioceptive control, they then attempted to actively relocate their head to 50% of the previously achieved maximal position with eyes closed. Both ROM and the deviation from the target position were recorded. Each measurement was repeated three times, and the average values were used for analysis. Larger deviations indicated reduced JPS, while smaller deviations indicated better proprioceptive control [29]. Cervical JPS was selected as an indirect indicator of sensorimotor function due to the documented functional interrelationship between the cervical and stomatognathic systems in individuals with TMD [9, 30].

Jaw Function was evaluated using the JFLS‐20, a component of the DC/TMD Axis II assessment. The scale consists of 20 items assessing functional limitations over the preceding month. Each item is scored on an 11‐point Likert scale ranging from 0 (no limitation) to 10 (severe limitation). The items are grouped into three subdomains: mastication, mandibular mobility and verbal/nonverbal communication. Subscale scores were calculated as the mean of the relevant items, and the total score was obtained by averaging the three subscale scores. The Turkish version of the JFLS‐20 has established validity and reliability [31].

Anxiety levels were assessed using the GAD‐7. This 7‐item questionnaire evaluates symptoms over the past 2 weeks, including feeling nervous, anxious, or on edge, inability to control worrying, excessive worry, restlessness, irritability, trouble relaxing, fear that something awful might happen. Each item is rated on a four‐point scale: 0 = not at all, 1 = several days, 2 = more than half the days, and 3 = nearly every day, yielding a total score from 0 to 21. Total scores of 5, 10 and 15 are cutoff points for mild, moderate, and severe anxiety, respectively. The Turkish version of the tool has been validated by Gündüz et al. [32].

Muscle pressure pain thresholds (PPTs) were measured using a Baseline Dolorimeter (Fabrication Enterprises, White Plains, USA) to guide the application of manual therapy. Pressure was applied to trigger points at a level corresponding to the participant's discomfort threshold, without exceeding pain tolerance. PPT values were used to standardize treatment pressure but were not analyzed as study outcomes.

### 2.7 Outcomes

Primary outcomes included mandibular mobility (mouth opening, right and left excursions, retrusion and protrusion), cervical JPS and jaw function. Anxiety levels were evaluated as a secondary outcome.

### 2.8 Statistical Analysis

Statistical analyses were performed using IBM SPSS Statistics version 25.0 (IBM Corp., Armonk, NY). Continuous variables were reported as mean ± standard deviation and categorical variables as frequencies. Normality of data distribution was assessed using the Shapiro–Wilk test, considering the relatively small sample size in each group (*n* = 19). The results indicated that the majority of outcome variables did not follow a normal distribution (*p* < 0.05). Therefore, nonparametric statistical methods were used for all analyses. Baseline comparisons between groups were performed using the Kruskal–Wallis test for continuous variables (age and disease duration), and the chi‐squared test for categorical variables (sex and TMD severity). Within‐group pre‐ and post‐treatment comparisons were conducted using the Wilcoxon Signed‐Rank test. Between‐group comparisons of change scores (Δ) were performed using the Kruskal–Wallis test. When significant differences were identified, Bonferroni‐adjusted pairwise comparisons were conducted.

Effect sizes were calculated to complement *p*‐values and to quantify the magnitude of observed effects. For within‐group comparisons, effect size (*r*) was calculated as *r* = *Z*/√*N*, where *Z* is the standardized test statistic from the Wilcoxon signed‐rank test and *N* is the total number of observations. For between‐group comparisons, eta squared (*η* ^2^) was calculated from the Kruskal–Wallis *H* statistic using *η* ^2^ = (*H* − k + 1)/(*N* − k), where *H* is the test statistic, *k* is the number of groups, and *N* is the total sample size. *η* ^2^ values of 0.01, 0.06 and 0.14 were considered as small, medium and large effect sizes, respectively [33]. Following Cohen's guidelines, *r* values of 0.1, 0.3 and 0.5 were interpreted as small, medium and large effect sizes, respectively [34]. The level of statistical significance was set at *p* < 0.05.

## 3 Results

**FIGURE 1:**
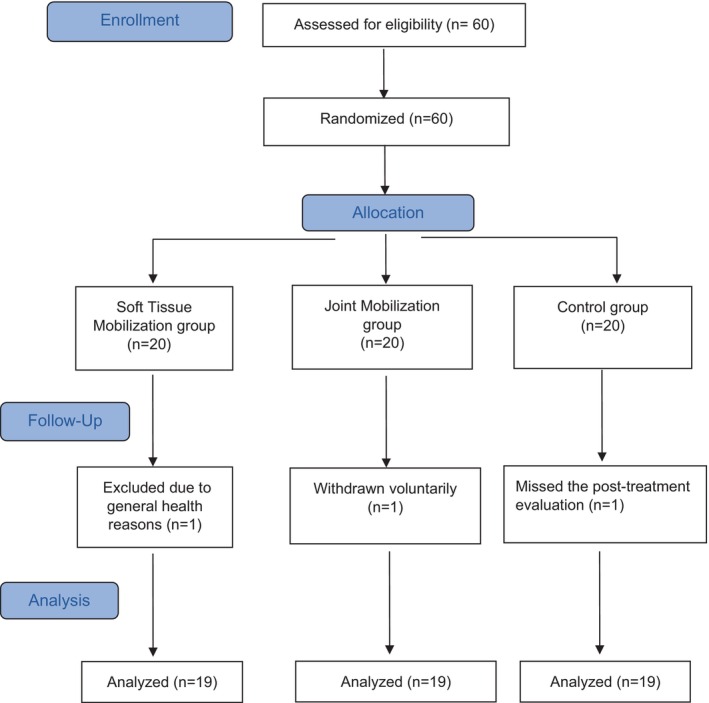
Study flow chart.

Initially, 60 patients were randomly allocated to the STM, JM, and CG groups (*n* = 20 per group). During the study, one participant in the STM group was excluded due to general health reasons, one participant in the JM group withdrew voluntarily, and one participant in the CG did not attend the post‐treatment assessment. Consequently, the study was completed with 57 individuals (*n* = 19 per group). The study flow diagram is presented in Figure 1.

The groups were comparable in terms of sex distribution, age, disease duration, and baseline TMD severity (*p* > 0.05) (Table 1).

**TABLE 1:** Demographic and baseline clinical characteristics of participants.

	STM group (*n* = 19)	JM group (*n* = 19)	CG (*n* = 19)	*p*
Sex				
Female, *n*	17	17	17	0.99
Male, *n*	2	2	2	
Age (years)	46.52 ± 13.29	43.00 ± 10.52	43.47 ± 11.26	0.286
Disease duration (years)	11.42 ± 4.43	11.26 ± 3.64	10.78 ± 4.00	0.843
TMD severity (FAI)				0.777
Mild, *n*	1	1	3	
Moderate, *n*	2	2	2	
Severe, *n*	16	16	14	

*Note:* Values are presented as number of participants (*n*, frequency) or mean ± standard deviation (SD). TMD severity is presented as number of participants per category.

Abbreviations: CG, control group; FAI, Fonseca Anamnestic Index; JM, joint mobilization; STM, soft tissue mobilization; TMD, temporomandibular disorder.

### 3.1 Mandibular Mobility

Assessment of mandibular mobility showed significant post‐treatment changes in both intervention groups across all measured parameters. In the STM group, significant improvements were observed in mouth opening, right and left excursions, retrusion and protrusion (*p* < 0.05), with effect sizes ranging from medium to large (*r* = 0.480–0.704). In the JM group, all parameters showed significant improvements (*p* < 0.05), with large effect sizes (*r* = 0.538–0.808). In the control group, no significant improvements were observed in right excursion (*p* = 0.596), left excursion (*p* = 0.305), or retrusion (*p* = 0.059), while mouth opening (Δ = −1.62, *p* = 0.024, *r* = 0.518) and protrusion significantly decreased (Δ = −0.36, *p* = 0.035, *r* = 0.484). Between‐group comparisons revealed significant differences between both intervention groups and the control group (*p* ≤ 0.006), with large effect sizes (*η* ^2^ = 0.153–0.501) (Table 2).

**TABLE 2:** Mandibular mobility before and after intervention by group, within‐group changes and between‐group comparisons.

Variable	STM group (*n* = 19)		Within‐group change (Δ)	*p* ^1^	*r*	JM group (*n* = 19)		Within‐group change (Δ)	*p* ^1^	*r*	CG (*n* = 19)		Within‐group change (Δ)	*p* ^1^	*r*	*p* ^2^	*η* ^2^
	Pre	Post				Pre	Post				Pre	Post					
Mouth opening	39 ± 9.28	43.84 ± 5.65	4.84 ± 5.41	0.002*	0.704	33.95 ± 7.55	41.16 ± 4.35	7.21 ± 5.94	0.001*	0.808	38.68 ± 5.56	37 ± 5.98	−1.62 ± 3.14	0.024*	0.518	< 0.001^a,b^	0.501
Right excursion	7.16 ± 2.22	8.05 ± 2.01	0.89 ± 1.55	0.031*	0.494	6.63 ± 2.5	8.42 ± 1.87	1.78 ± 2.04	0.003*	0.681	6.53 ± 1.9	6.42 ± 2.22	−0.10 ± 1.32	0.596	0.121	0.001^b^	0.204
Left excursion	7.11 ± 2.56	8.16 ± 1.98	1.05 ± 1.54	0.011*	0.581	6.63 ± 3.2	8.32 ± 2.38	1.68 ± 2.21	0.004*	0.669	7.11 ± 2.16	6.89 ± 2.26	−0.21 ± 0.85	0.305	0.235	0.001^a,b^	0.221
Retrusion	3.11 ± 1.24	3.89 ± 1.56	0.78 ± 1.65	0.036*	0.480	2.53 ± 1.43	3.42 ± 1.92	0.89 ± 1.37	0.019*	0.538	2.42 ± 1.46	2.11 ± 1.37	−0.31 ± 0.74	0.059	0.433	0.006^a,b^	0.153
Protrusion	4.95 ± 2.53	6 ± 2.16	1.05 ± 1.47	0.007*	0.619	4.16 ± 1.74	5.68 ± 1.73	1.52 ± 1.46	0.002*	0.726	4.42 ± 1.57	4.05 ± 1.61	−0.36 ± 0.68	0.035*	0.484	< 0.001^a,b^	0.387

*Note:* Values are presented as mean ± SD in millimetres (mm). *p < 0.05. *p* ^1^: within‐group comparisons (Wilcoxon Signed‐Rank Test); *p* ^2^: between‐group comparisons based on change scores (Kruskal–Wallis test). Significant pairwise comparisons were determined using Bonferroni‐adjusted post hoc tests: *p* ^a^ = STM group versus CG, *p* ^b^ = JM group versus CG. *r* values were calculated from *Z* statistics using *r* = *Z*/√*N*. *η* ^2^ values were calculated from *H* statistics using *η* ^2^ = (*H* − *k* + 1)/(*N* − *k*). Bold values indicate statistically significant results (*p* < 0.05).

Abbreviations: CG, control group; JM, joint mobilization; STM, soft tissue mobilization.

### 3.2 Cervical Proprioception

Assessment of cervical JPS showed significant improvements (with large effect sizes) in the STM group for flexion (*p* = 0.019, *r* = 0.538), extension (*p* = 0.008, *r* = 0.604), and left rotation (*p* = 0.020, *r* = 0.535). In the JM group, a significant improvement was observed only in flexion (*p* = 0.004), with a large effect size (*r* = 0.660). No significant changes were observed in the control group (all *p* > 0.05). Between‐group comparisons of change scores revealed a significant difference in flexion between the STM group and the control group (*p* = 0.023), with a medium effect size (*η* ^2^ = 0.103). No other between‐group comparisons were statistically significant (all *p* > 0.05), with effect sizes ranging from *η* ^2^ = 0.004 (small) to 0.130 (medium) (Table 3).

**TABLE 3:** Cervical joint position sense before and after intervention by group, within‐group changes and between‐group comparisons.

Variable	STM group (*n* = 19)		Within‐group change (Δ)	*p* ^1^	*r*	JM group (*n* = 19)		Within‐group change (Δ)	*p* ^1^	*r*	CG (*n* = 19)		Within‐group change (Δ)	*p* ^1^	*r*	*p* ^2^	*η* ^2^
	Pre	Post				Pre	Post				Pre	Post					
Flexion (°)	5.53 ± 4.97	1.32 ± 2.81	−4.21 ± 6.51	0.019*	0.538	4.47 ± 4.05	0.53 ± 1.58	−3.94 ± 4.27	0.004*	0.660	1.84 ± 3.42	1.05 ± 2.68	−0.78 ± 2.50	0.18	0.307	0.023*^a^	0.103
Extension (°)	4.42 ± 4.05	1.32 ± 2.26	−3.10 ± 4.52	0.008*	0.604	1.84 ± 3.8	1.32 ± 2.26	−0.52 ± 4.37	0.557	0.134	2.11 ± 3.84	1.32 ± 2.81	−0.78 ± 3.82	0.408	0.189	0.130	0.039
Right lateral flexion (°)	2.53 ± 3.44	1.05 ± 2.68	−1.47 ± 3.67	0.119	0.357	2 ± 2.96	1.05 ± 2.09	−0.94 ± 2.19	0.066	0.421	0 ± 0	0.26 ± 1.15	0.26 ± 1.14	0.317	0.229	0.113	0.044
Left lateral flexion (°)	1.84 ± 3.42	0.53 ± 1.58	−1.31 ± 3.66	0.129	0.348	1.32 ± 2.26	1.05 ± 2.09	−0.26 ± 3.10	0.705	0.086	0.26 ± 1.15	0.26 ± 1.15	0.00 ± 0.00	0.99	0.000	0.489	0.000
Right Rotation (°)	2.89 ± 4.19	1.32 ± 2.81	−1.57 ± 5.54	0.201	0.293	2.37 ± 4.21	0.53 ± 1.58	−1.84 ± 3.80	0.059	0.433	0.26 ± 1.15	0.53 ± 2.29	0.26 ± 2.62	0.655	0.102	0.328	0.004
Left Rotation (°)	2.63 ± 3.48	0.79 ± 2.51	−1.84 ± 2.98	0.02*	0.535	1.58 ± 3.36	0.26 ± 1.15	−1.31 ± 2.80	0.059	0.433	0.53 ± 2.29	0.79 ± 2.51	0.26 ± 2.62	0.655	0.102	0.072	0.06

*Note:* Values represent absolute error in reproducing target joint angles and are reported as mean ± SD. *p < 0.05. *p* ^1^: within‐group comparisons (Wilcoxon Signed‐Rank Test); *p* ^2^: between‐group comparisons based on change scores (Kruskal–Wallis test). Significant pairwise comparisons were determined using Bonferroni‐adjusted post hoc tests: *p* ^a^ = STM group versus CG, *p* ^b^ = JM group versus CG. *r* values were calculated from *Z* statistics using *r* = *Z*/√*N*. *η* ^2^ values were calculated from *H* statistics using *η* ^2^ = (*H* − *k* + 1)/(*N* − *k*). Bold values indicate statistically significant results (*p* < 0.05).

Abbreviations: CG, control group; JM, joint mobilization; STM, soft tissue mobilization.

### 3.3 Jaw Function and Anxiety

Assessment of jaw function using the JFLS‐20 showed significant improvements in all subdomains (mastication, mobility, and communication) and the total score in both the STM and JM groups (*p* < 0.001), with large within‐group effect sizes (*r* = 0.795–0.878). In contrast, the control group showed significant worsening in mastication, mobility, and total JFLS‐20 score, with large effect sizes (*r* = 0.514–0.616). No significant change was observed in communication score (*p* = 0.105, *r* = 0.371).

Between‐group comparisons revealed significant differences in favour of both intervention groups compared to the control group across all functional outcomes, with large effect sizes (*η* ^2^ = 0.432–0.654). No significant differences were observed between the STM and JM groups (*p* > 0.05).

Regarding anxiety levels (GAD‐7), both intervention groups showed significant reductions (STM: *r* = 0.783; JM: *r* = 0.791; both, *p* = 0.001), whereas the control group showed a significant increase (*r* = 0.462, *p* = 0.044). Between‐group comparisons revealed a significant difference in GAD‐7 change scores, favouring the intervention groups over the control group (*η* ^2^ = 0.432, *p* < 0.001) (Table 4).

**TABLE 4:** JFLS‐20 and GAD‐7 scores before and after intervention by group, within‐group changes and between‐group comparisons.

Variable	STM (*n* = 19)		Within‐group change (Δ)	*p* ^1^	*r*	JM (*n* = 19)		Within‐group change (Δ)	*p* ^1^	*r*	CG (*n* = 19)		Within‐group change (Δ)	*p* ^1^	*r*	*p* ^2^	*η* ^2^
	Pre	Post				Pre	Post				Pre	Post					
JFLS‐20 Mastication (score)	3.66 ± 1.38	1.26 ± 0.90	−2.40 ± 1.57	< 0.000*	0.863	3.39 ± 1.72	0.68 ± 0.76	−2.71 ± 1.67	< 0.000*	0.878	3.22 ± 1.24	3.41 ± 1.20	0.19 ± 0.35	0.025*	0.514	< 0.000*^a,b^	0.595
JFLS‐20 mobility (score)	5.21 ± 1.69	1.53 ± 1.22	−3.68 ± 2.07	< 0.000*	0.869	4.65 ± 2.65	0.58 ± 0.77	−4.06 ± 2.59	< 0.000*	0.855	3.94 ± 1.95	4.61 ± 1.86	0.67 ± 1.14	0.007*	0.616	< 0.000*^a,b^	0.593
JFLS‐20 communication (score)	3.51 ± 2.61	1.03 ± 1.53	−2.47 ± 2.48	0.001*	0.795	3.14 ± 2.24	0.55 ± 0.92	−2.58 ± 2.04	< 0.000*	0.840	3.39 ± 2.32	3.64 ± 2.07	0.24 ± 0.81	0.105	0.371	< 0.000*^a,b^	0.456
JFLS‐20 Total	4.12 ± 1.32	1.21 ± 0.98	−2.90 ± 1.53	< 0.000*	0.878	3.68 ± 1.80	0.58 ± 0.48	−3.10 ± 1.66	< 0.000*	0.878	3.48 ± 1.55	3.85 ± 1.41	0.36 ± 0.62	0.009*	0.596	< 0.000*^a,b^	0.654
GAD‐7 (score)	9.95 ± 5.82	4.74 ± 5.23	−5.21 ± 4.61	0.001*	0.783	9.11 ± 5	4.26 ± 2.94	−4.84 ± 4.23	0.001*	0.791	8.95 ± 5.35	10.11 ± 6	1.15 ± 2.45	0.044*	0.462	< 0.000*^a,b^	0.432

*Note:* Values are presented as mean ± SD. *p < 0.05. *p* ^1^: within‐group comparisons (Wilcoxon Signed‐Rank Test); *p* ^2^: between‐group comparisons based on change scores (Kruskal–Wallis test). Significant pairwise comparisons were determined using Bonferroni‐adjusted post hoc tests: *p* ^a^ = SM group versus CG, *p* ^b^ = JM group versus CG. *r* values were calculated from *Z* statistics using *r* = *Z*/√*N*. *η* ^2^ values were calculated from *H* statistics using *η* ^2^ = (*H* − *k* + 1)/(*N* − *k*).

Abbreviations: CG, control group; GAD‐7, Generalized Anxiety Disorder‐7; JFLS‐20, Jaw Functional Limitation Scale‐20; JM, joint mobilization; SM, soft tissue mobilization.

## 4 Discussion

In this study investigating the effects of soft tissue mobilization and joint mobilization on mandibular mobility, cervical JPS, jaw function and anxiety in individuals with RA and TMD, both manual therapy techniques resulted in significant improvements in most assessed outcomes compared with the control group. Joint mobilization demonstrated significant improvements across all directions of mandibular movement. STM was associated with significant improvements in flexion, extension, and left rotation in cervical JPS, whereas JM improved cervical JPS only in flexion. Both interventions significantly improved jaw function and reduced anxiety levels, while the control group demonstrated deterioration in mouth opening, protrusion, and anxiety over time.

Regarding mandibular mobility, both STM and JM produced significant improvements in mouth opening, lateral excursions, retrusion, and protrusion. The JM group showed consistent improvements across all mandibular movement directions, with higher effect sizes observed in some parameters compared to the STM group (JM: *r* = 0.538–0.808 vs. STM: *r* = 0.480–0.704). These findings are consistent with previous studies by Barone et al. and Grondin et al., which reported the superior effect of JM on mandibular mobility due to its mechanical advantage in addressing capsular restrictions [35, 36]. The pronounced gains observed in the JM group are also in line with Potewiratnanond et al., who reported significant short‐term improvements following TMJ mobilization [16]. Joint‐specific mobilization targets capsular restrictions, facilitates glide between joint surfaces, and enhances mechanical elasticity. The observed gains particularly in the JM group parallel the corrective effects of joint mobilization on TMJ arthrokinematics described in a previous review [37]. These findings support the potential value of JM for restoring mandibular movements, particularly in patients exhibiting RA‐associated capsular and synovial changes. The deterioration observed in mouth opening and protrusion in the control group may reflect natural variability of TMD symptoms combined with the absence of active therapeutic intervention, which may contribute to persistent muscle guarding and reduced joint mobility over time in susceptible individuals.

Nevertheless, the significant improvements observed in the STM group indicate that soft tissue‐based interventions may also contribute meaningfully to mandibular mobility. Gębska et al. reported that STM combined with therapeutic exercises increased MMO, consistent with the improvements observed in our STM group [38]. Rezaie et al. similarly demonstrated the superiority of manual interventions over routine treatments, which aligns with our findings that both intervention groups outperformed the control in all mandibular mobility parameters [39]. Collectively, these findings indicate that manual therapy, whether focused on joint structures or soft tissues, can effectively improve mandibular mobility in individuals with RA‐related TMD, although JM appears to produce a more consistent pattern of improvement across mandibular movements.

Current literature confirms that cervical JPS is significantly impaired in individuals with TMD and represents a valid proxy for sensorimotor control within the stomatognathic system [9, 30]. Therefore, cervical JPS assessment was selected to comprehensively evaluate jaw‐neck functional interactions in the present study. However, direct evidence regarding cervical JPS changes following TMJ manual therapy remains limited, with most available knowledge extrapolated from cervical spine studies, broader multimodal TMD reviews, and mechanistic reasoning rather than TMJ‐specific proprioceptive investigations [40]. A notable finding of our study was the broader improvement in cervical JPS observed in the STM group. Significant gains were detected in flexion, extension, and left rotation following STM, whereas JM improved cervical JPS only in flexion. This finding aligns with Yüzbaşıoğlu et al., who reported that in individuals with myogenous TMD, co‐activation of the sternocleidomastoid and masseter muscles during functional activities, combined with abnormal cervical posture, may disrupt proprioceptive afferent input and adversely affect JPS [30]. The greater improvement observed following STM supports this mechanism. Tension and trigger points arising from affected muscular and fascial structures may impair proprioception, and the applied soft tissue techniques may reduce muscular hyperactivity while enhancing sensorimotor control.

La Touche et al. emphasized the importance of cervical motor control in jaw function [41]. Our findings suggest that manipulation of muscular and fascial structures may regulate afferent input more effectively, thereby contributing to improved proprioception. This interpretation is further supported by studies indicating that cervical and TMJ manual therapies, including soft tissue release, may provide nociceptive and proprioceptive modulation through trigeminocervical integration [39]. Similarly, Miçooğulları et al. reported that exercises targeting the scapulothoracic region improved both TMJ and cervical JPS, further emphasizing the role of sensorimotor integration across the cranio‐cervico‐mandibular system and supporting the concept that interventions beyond the TMJ may influence proprioceptive control through interconnected neuromuscular pathways [42]. STM likely reduced muscular hyperactivity and fascial tension, thereby enhancing sensorimotor control and proprioceptive afferent input.

Previous research also suggests that TMJ manual therapy may positively influence the cervical region. Sulowska‐Daszyk et al. reported that a single session of STM improved both mandibular and cervical spine mobility [43]. Grondin et al. observed reductions in cervical movement restrictions following isolated TMJ manual therapy [36], while Rezaie et al. demonstrated benefits in both jaw and cervical function after combined TMJ and upper cervical manual therapy [39]. Similarly, Liberato et al. emphasized that interventions targeting the TMJ may indirectly enhance cervical proprioception and function through trigeminocervical connections [44]. Collectively, these findings support the potential for STM to improve cervical JPS via neurophysiological and biomechanical links.

Regarding jaw function, both intervention groups demonstrated significant improvements in all JFLS‐20 subdomains and total scores, with no statistically significant difference between STM and JM. These findings are consistent with previous literature indicating that manual therapy has positive effects on functional limitations in TMD [37]. The lack of superiority between intervention groups suggests that both techniques may improve jaw function through shared physiological mechanisms, including reduction of pathological tension in joint capsules and surrounding tissues, improved muscular efficiency, and restoration of impaired proprioceptive feedback [15]. These results align with prior studies demonstrating that manual therapy, regardless of the specific tissue targeted, enhances TMJ function and mastication performance [38, 41, 44].

Anxiety levels in our study were assessed using the GAD‐7 scale. Both STM and JM groups demonstrated significant reductions in anxiety, whereas the control group showed an increase in GAD‐7 scores post‐intervention. These findings are consistent with the reciprocal relationship between emotional tension, muscle hyperactivity and TMJ pain mechanisms. Previous studies have highlighted that psychological stress may exacerbate musculoskeletal symptoms and that psychosocial factors encompassed by Axis II, including anxiety and somatization, play a critical role in modulating pain perception, muscle sensitivity, and functional performance [45, 46]. The similar reductions in anxiety observed in both intervention groups may reflect the neuromodulatory effects of manual therapy, including decreased muscle tone and modulation of nociceptive pathways, potentially alleviating the overall psychological burden on patients [47]. The worsening observed in the control group may be explained by symptom progression in the absence of treatment as well as nonspecific psychological factors, including reduced treatment expectancy or nocebo‐related responses [48].

The functional gains observed in our study should be interpreted in the context of the systemic nature of RA, including disease duration, inflammatory burden, and pharmacological therapies. Chronic inflammation in RA is associated with synovial proliferation and fibrotic changes in joint capsules, which may directly affect TMJ mechanics [49]. In individuals with long‐standing disease, systemic inflammatory mediators such as TNF‐α and IL‐6 can enhance pain sensitization and impair tissue repair processes [50]. In addition, corticosteroids and disease‐modifying antirheumatic drugs (DMARDs) may influence connective tissue collagen structure, potentially modulating the response to manual therapy interventions [13]. Although all participants were in remission, the interaction between these RA‐related systemic factors and the neuromuscular and mechanical effects of manual therapy may have contributed to the observed clinical outcomes.

### 4.1 Limitations

A limitation of this study is that, although TMD was diagnosed using the Diagnostic Criteria for Temporomandibular Disorders (DC/TMD), patients were not further stratified according to DC/TMD diagnostic subgroups. Therefore, potential differences in treatment response across pain‐related and intra‐articular TMD subtypes could not be evaluated. Future studies with subgroup‐specific analyses are needed to better clarify treatment effects across distinct DC/TMD diagnostic categories. Second, the study was conducted at a single centre, which may limit the generalizability of the findings to the broader TMD population. Third, proprioception was assessed indirectly through cervical JPS rather than by direct measurement of TMJ JPS. Future studies may benefit from incorporating direct TMJ proprioceptive assessments to provide a more localized evaluation. Fourth, although TMD is known to be associated with head and neck posture, postural characteristics were not assessed in the present study. Future investigations incorporating postural evaluation may provide more robust findings. Fifth, the absence of any intervention in the control group, such as muscle relaxation techniques, limits the ability to distinguish whether observed symptom changes are due to natural fluctuations or potentially influenced by a nocebo effect.

Furthermore, the effectiveness of the interventions was evaluated only immediately after treatment, without long‐term follow‐up. In chronic and progressive conditions such as RA, evaluating the persistence of functional gains is clinically important. Regarding psychosocial outcomes, patients' positive treatment expectations or placebo effects may have influenced GAD‐7 scores. Finally, RA‐specific factors such as disease duration and systemic inflammatory burden were not analysed in depth. Future placebo‐controlled longitudinal studies are warranted to further validate and extend these findings.

## 5 Conclusion

Both manual therapy techniques effectively improved mandibular mobility, cervical joint position sense, jaw function, and anxiety levels in individuals with RA and TMD. Joint mobilization yielded greater improvements in mandibular mobility, whereas soft tissue mobilization demonstrated broader benefits for cervical joint position sense. These findings suggest that the two approaches may contribute to temporomandibular rehabilitation through distinct therapeutic mechanisms. As non‐invasive interventions, both may serve as clinically applicable treatment options for TMD in individuals with rheumatoid arthritis. Future studies should further investigate the mechanisms underlying these effects and evaluate their long‐term sustainability.

## Author Contributions

**Kübra Aktaş:** conceptualization, writing – review and editing, supervision, data curation, methodology. **Hakan Polat:** writing – review and editing, writing – original draft, data curation, supervision, methodology. **Tuba Maden:** writing – original draft, writing – review and editing. **Burhan Fatih Koçyiğit:** data curation.

## Conflicts of Interest

The authors declare no conflicts of interest.

## Data Availability

The data that support the findings of this study are available on request from the corresponding author. The data are not publicly available due to privacy or ethical restrictions.

## Appendix 1

Soft tissue mobilization

**Table:**

Technique	Technical details
Trigger points—Masseter muscle	The patient was positioned supine; and the physical therapist was seated. Trigger points in the masseter muscle were identified based on assessment findings. The muscle was grasped both intraorally and extraorally in a hook position, and direct pressure was applied for 60–90 s, allowing the tissue to relax. Pressure was maintained at the patient's discomfort threshold without causing pain. When tissue relaxation was felt under the therapist's finger, pressure was decreased and then gradually increased again. This procedure was repeated three times on both the right and left masseter muscles.
Trigger points—Temporalis muscle	The patient was supine and the therapist seated. Direct perpendicular pressure was applied to trigger points in the temporalis muscle for 60–90 s, allowing tissue relaxation. Pressure was maintained at the patient's discomfort threshold without causing pain. When tissue relaxation was perceived, pressure was decreased and then gradually increased again. The procedure was repeated three times on each side.
Trigger points—Medial pterygoid muscle	With the patient supine and the therapist seated, direct intraoral pressure was applied to trigger points in the medial pterygoid muscle for 60–90 s, allowing tissue relaxation. Pressure was kept at the patient's discomfort threshold without causing pain. When tissue relaxation was felt, pressure was decreased and then gradually increased again. The procedure was repeated three times on each side.
Trigger points—Lateral pterygoid muscle	The patient was supine, and the therapist applied direct intraoral pressure to trigger points in the lateral pterygoid muscle for 60–90 s, allowing tissue relaxation. Pressure was maintained at the patient's discomfort threshold without causing pain. Upon tissue relaxation, pressure was decreased and then gradually increased again. This procedure was repeated three times on both sides.
Trigger points—digastric muscle	With the patient supine, vertical direct pressure was applied to trigger points in the digastric muscle for 60–90 s, allowing tissue relaxation. Pressure was maintained at the patient's discomfort threshold without causing pain. When tissue relaxation was perceived, pressure was decreased and then gradually increased again. The procedure was repeated three times on each side.
Myofascial release—Masseter muscle	The patient was positioned supine. The therapist applied pressure starting at the origin of the masseter muscle, gradually deepening pressure along the fascia towards the insertion in a sliding motion. Myofascial release was applied for approximately 90 s on both sides.
Myofascial release—Temporalis muscle	With the patient supine, the therapist applied pressure from the origin of the temporalis muscle along the fascia towards the insertion in a sliding motion. Myofascial release was applied for approximately 90 s on both sides.
Myofascial release—Digastric muscle	The patient was supine. Pressure was applied from the origin of the digastric muscle along the fascia towards the insertion points in a sliding motion. Myofascial release was applied for approximately 90 s on both sides.
Myofascial release—Mandibular fascia	The patient was supine. The therapist placed their fingertips beneath the chin on the floor of the mouth fascia and performed pulling movements forward and inward, then laterally and towards the jaw. The procedure was repeated 2–5 times until all tension was released, lasting approximately 90 s.
Muscle Energy technique—Mandibular opening	With the patient supine, the therapist placed their thumbs on the mandibular symphysis and supported the jaw with their other fingers. The patient was instructed to open the mouth to the point of limitation, after which submaximal resistance was applied for 10 s while attempting closure. Following relaxation, the patient opened the mouth further. This procedure was repeated three times.
Muscle energy technique—Lateral mandibular contraction to the left	The patient was supine and instructed to press the chin to the left with submaximal force for 10 s up to the point of limitation. After relaxation, right lateral flexion was increased. The procedure was repeated three times.
Muscle energy technique—Lateral mandibular contraction to the right	The patient was supine and instructed to press the chin to the right with submaximal force for 10 s up to the point of limitation. After relaxation, left lateral flexion was increased. The procedure was repeated three times.

## Appendix 2

Joint mobilization

**Table:**

Technique	Technical details
Caudal displacement	The patient is positioned supine, with the therapist standing beside them. The therapist's thumb is positioned parallel to the patient's mouth and aligned with the posterior‐inferior molar teeth. Traction is applied in the caudal (inferior) direction. Mobilization is performed at Grade II–III intensity in a rhythmic manner for 30 s per set, repeated three times
Anterior displacement	With the patient supine and the therapist standing beside them, the therapist's thumb is hooked over the lower four anterior teeth. Traction is applied anteriorly (forward). Mobilization is performed at Grade II–III intensity, with controlled pressure applied to the joint limit. The procedure is performed rhythmically for 30 s per set, repeated three times.
Mediolateral translation	The patient is positioned supine, with the therapist standing beside them. The therapist's thumbs are positioned on top of each other on the mandibular condyle of the side being treated. The condyle is mobilized medially. Mobilization is performed at Grade II intensity for 10–15 repetitions.
Ventro‐caudal translation	With the patient supine and the therapist standing beside them, the therapist's thumb is positioned parallel to the patient's mouth and aligned with the posterior‐inferior molar teeth. A ventro‐caudal translation is applied, combining anterior (ventral) and inferior (caudal) sliding movement. Mobilization is performed at Grade II–III intensity for 10–20 s per repetition, repeated three times.
